# *N*-(12-Amino-9,10-di­hydro-9,10-ethano­anthracen-11-yl)-4-methyl­benzene­sulfonamide

**DOI:** 10.1107/S1600536814002189

**Published:** 2014-02-05

**Authors:** Joel T. Mague, Alaa A.-M. Abdel-Aziz, Adel S. El-Azab, Magda A. El-Sherbeny

**Affiliations:** a Department of Chemistry, Tulane University, New Orleans, LA 70118, USA; b Department of Pharmaceutical Chemistry, College of, Pharmacy, King Saud University, Riyadh 11451, Saudi Arabia; c Department of Medicinal Chemistry, Faculty of Pharmacy, University of Mansoura, Mansoura 35516, Egypt; d Department of Organic Chemistry, Faculty of Pharmacy, Al-Azhar University, Cairo 11884, Egypt

## Abstract

The title compound, C_23_H_22_N_2_O_2_S, crystallizes with the 4-methyl­benzene­sulfonamide entity oriented towards the center of the bridgehead C atoms with a C—N—S—C torsion angle of −61.3 (2)°. The mol­ecule features an intra­molecular N—H⋯N hydrogen bond. Weak C—H⋯O and C—H⋯π inter­actions aid in forming the three-dimensional supra­molecular structure.

## Related literature   

For chiral ligand devlopment, see: Abdel-Aziz *et al.* (2000 ▶, 2001 ▶, 2004 ▶); Matsunaga *et al.* (2005 ▶); Seo *et al.* (2001 ▶). For similar compounds and applications, see: Yamakuchi *et al.* (2005 ▶); Matsunaga *et al.* (2005 ▶); Abdel-Aziz *et al.* (2004 ▶). For the synthesis of the title compound, see: Matsunaga *et al.* (2005 ▶).
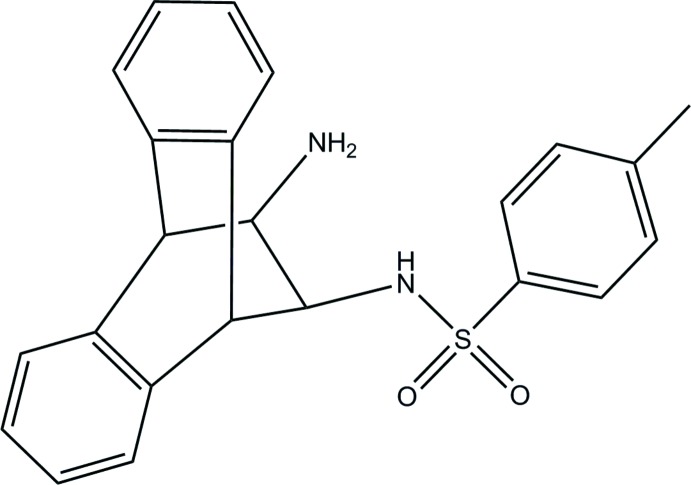


## Experimental   

### 

#### Crystal data  

C_23_H_22_N_2_O_2_S
*M* *_r_* = 390.49
Monoclinic, 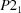

*a* = 8.9362 (2) Å
*b* = 6.8766 (2) Å
*c* = 15.5039 (4) Å
β = 91.540 (1)°
*V* = 952.38 (4) Å^3^
*Z* = 2
Cu *K*α radiation
μ = 1.68 mm^−1^
*T* = 100 K
0.25 × 0.13 × 0.03 mm

#### Data collection  

Bruker D8 VENTURE PHOTON 100 CMOS diffractometer
Absorption correction: multi-scan (*SADABS*; Bruker, 2012 ▶) *T*_min_ = 0.83, *T*_max_ = 0.95
16018 measured reflections
3531 independent reflections
3255 reflections with *I* > 2σ(*I*)
*R*_int_ = 0.035

#### Refinement  

*R*[*F* ^2^ > 2σ(*F* ^2^)] = 0.028
*wR*(*F* ^2^) = 0.066
*S* = 1.07
3531 reflections
282 parameters
1 restraint
H atoms treated by a mixture of independent and constrained refinement
Δρ_max_ = 0.17 e Å^−3^
Δρ_min_ = −0.21 e Å^−3^
Absolute structure: Flack (1983 ▶), 1582 Friedel pairs
Absolute structure parameter: 0.036 (13)

### 

Data collection: *APEX2* (Bruker, 2012 ▶); cell refinement: *SAINT* (Bruker, 2012 ▶); data reduction: *SAINT*; program(s) used to solve structure: *SHELXS97* (Sheldrick, 2008 ▶); program(s) used to refine structure: *SHELXL97* (Sheldrick, 2008 ▶); molecular graphics: *DIAMOND* (Brandenburg & Putz, 2012 ▶); software used to prepare material for publication: *SHELXTL* (Bruker, 2012 ▶).

## Supplementary Material

Crystal structure: contains datablock(s) global, I. DOI: 10.1107/S1600536814002189/tk5292sup1.cif

Structure factors: contains datablock(s) I. DOI: 10.1107/S1600536814002189/tk5292Isup2.hkl

CCDC reference:

Additional supporting information:  crystallographic information; 3D view; checkCIF report

## Figures and Tables

**Table 1:** Hydrogen-bond geometry (Å, °) *Cg*1 is the centroid of the C3–C8 benzene ring.

*D*—H⋯*A*	*D*—H	H⋯*A*	*D*⋯*A*	*D*—H⋯*A*
N1—H1*N*⋯N2	0.90 (2)	2.04 (2)	2.592 (2)	117.9 (17)
C11—H11⋯*Cg*1^i^	0.95	2.87	3.712 (2)	149
C5—H5⋯O1^ii^	0.95	2.58	3.269 (2)	129
C12—H12⋯O1^iii^	0.95	2.52	3.446 (2)	166
C23—H23*A*⋯O1^iv^	0.98	2.51	3.259 (3)	133

Symmetry codes: (i) 
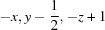
; (ii) 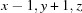; (iii) 
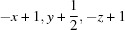
; (iv) 
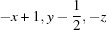
.

**Fig. 1.**
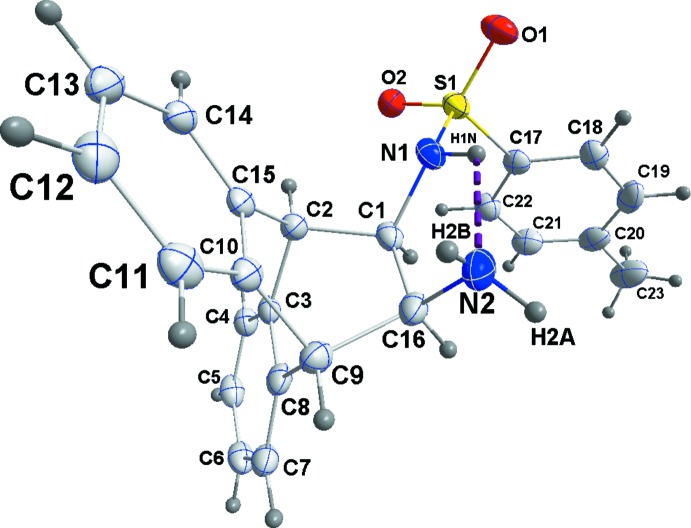
Perspective view of the title molecule showing the intramolecular hydrogen bond. Displacement ellipsoids are drawn at the 50% probability level.

**Fig. 2.**
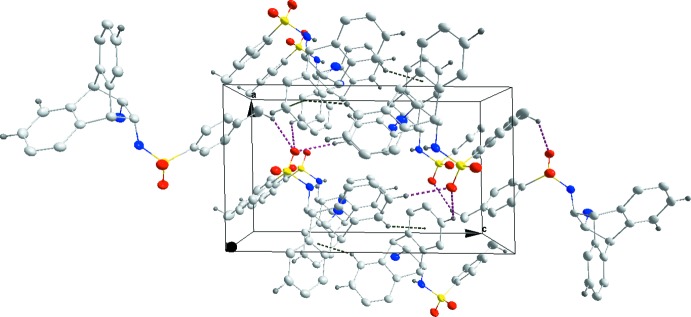
Packing diagram viewed down b with intermolecular interactions shown as dotted lines (C—H···O, purple; C—H···π, green).

## supplementary crystallographic information

### 2.1. Synthesis and crystallization

The title compound was prepared by the literature method (Matsunaga *et al.*, 2005) and recrystallized from CH_2_Cl_2_/EtOH as colourless plates.

### 2.2. Refinement

H-atoms attached to the bridgehead carbon atoms and to nitro­gen were located and refined. The remainder were placed in calculated positions (C—H = 0.95 - 0.98 Å) and included as riding contributions with isotropic displacement parameters 1.2–1.5 times those of the attached carbon atoms.

### 3. Results and discussion

The development of chiral ligands for asymmetric catalytic reactions is a subject of considerable inter­est in the field of asymmetric synthesis (Abdel-Aziz *et al.*, 2000, 2001, 2004; Matsunaga *et al.*, 2005; Seo *et al.*, 2001). As part of our onging program of drug design and discovery we report the structure of the title compound. Some applications of related compounds have been reported (Yamakuchi *et al.*, 2005; Matsunaga *et al.*, 2005; Abdel-Aziz *et al.*, 2004). The 4-methyl­benzene­sulfonamide entity is oriented towards the center of the bridgehead carbon atoms (C1, C16), partly due to the N1—H1N···N2 inter­action, as indicated by the C1—N1—S1—C17 torsion angle of -61.3 (2)°. The dihedral angle between the benzene ring (C17–C22) and the mean plane of the C1/C2/C9/C16 unit is 87.78 (7)°. The packing of the molecules is aided by weak C—H···O hydrogen bonds as well as a C—H···π inter­action between C11—H11 and the centroid of the C3–C8 ring at -*x*, -0.5 + *y*, 1 - *z* forming the three-dimensional supra­molecular structure (Table 1 and Fig. 2).

### Crystal data

**Table:**

C_23_H_22_N_2_O_2_S	*F*(000) = 412
*M**_r_* = 390.49	*D*_x_ = 1.362 Mg m^−^^3^
Monoclinic, *P*2_1_	Cu *K*α radiation, λ = 1.54178 Å
*a* = 8.9362 (2) Å	Cell parameters from 9974 reflections
*b* = 6.8766 (2) Å	θ = 2.9–69.8°
*c* = 15.5039 (4) Å	µ = 1.68 mm^−^^1^
β = 91.540 (1)°	*T* = 100 K
*V* = 952.38 (4) Å^3^	Plate, colourless
*Z* = 2	0.25 × 0.13 × 0.03 mm

### Data collection

**Table:**

Bruker D8 VENTURE PHOTON 100 CMOS diffractometer	3531 independent reflections
Radiation source: INCOATEC IµS micro-focus source	3255 reflections with *I* > 2σ(*I*)
Mirror monochromator	*R*_int_ = 0.035
Detector resolution: 10.4167 pixels mm^-1^	θ_max_ = 69.8°, θ_min_ = 2.9°
ω scans	*h* = −10→10
Absorption correction: multi-scan (*SADABS*; Bruker, 2012)	*k* = −8→8
*T*_min_ = 0.83, *T*_max_ = 0.95	*l* = −18→18
16018 measured reflections	

### Refinement

**Table:**

Refinement on *F*^2^	Secondary atom site location: difference Fourier map
Least-squares matrix: full	Hydrogen site location: inferred from neighbouring sites
*R*[*F*^2^ > 2σ(*F*^2^)] = 0.028	H atoms treated by a mixture of independent and constrained refinement
*wR*(*F*^2^) = 0.066	*w* = 1/[σ^2^(*F*_o_^2^) + (0.0302*P*)^2^ + 0.1321*P*] where *P* = (*F*_o_^2^ + 2*F*_c_^2^)/3
*S* = 1.07	(Δ/σ)_max_ = 0.001
3531 reflections	Δρ_max_ = 0.17 e Å^−^^3^
282 parameters	Δρ_min_ = −0.21 e Å^−^^3^
1 restraint	Absolute structure: Flack (1983), 1582 Friedel pairs
Primary atom site location: structure-invariant direct methods	Absolute structure parameter: 0.036 (13)

### Special details

**Table:**

Experimental. The diffraction data were obtained from 8 sets of 340 frames, each of width 0.5° in ω, collected at φ = 0.00, 90.00, 180.00 and 270.00° and at 2θ = -50.00 and -90.00° and 4 sets of 340 frames, each of width 0.5° in ω collected at 2θ = -90.00° and φ = 45.00, 135.00, 225.00 and 315.00°. The scan time was 20 sec/frame.
Geometry. All e.s.d.'s (except the e.s.d. in the dihedral angle between two l.s. planes) are estimated using the full covariance matrix. The cell e.s.d.'s are taken into account individually in the estimation of e.s.d.'s in distances, angles and torsion angles; correlations between e.s.d.'s in cell parameters are only used when they are defined by crystal symmetry. An approximate (isotropic) treatment of cell e.s.d.'s is used for estimating e.s.d.'s involving l.s. planes.
Refinement. Refinement of *F*^2^ against ALL reflections. The weighted *R*-factor *wR* and goodness of fit *S* are based on *F*^2^, conventional *R*-factors *R* are based on *F*, with *F* set to zero for negative *F*^2^. The threshold expression of *F*^2^ > σ(*F*^2^) is used only for calculating *R*-factors(gt) *etc*. and is not relevant to the choice of reflections for refinement. *R*-factors based on *F*^2^ are statistically about twice as large as those based on *F*, and *R*- factors based on ALL data will be even larger. H-atoms attached to the bridgehead carbon atoms and to nitrogen were located and refined. The remainder were placed in calculated positions (C—H = 0.95 - 0.98 Å) and included as riding contributions with isotropic displacement parameters 1.2 - 1.5 times those of the attached carbon atoms.

### Fractional atomic coordinates and isotropic or equivalent isotropic displacement parameters (Å^2^)

**Table:**

	*x*	*y*	*z*	*U*_iso_*/*U*_eq_	
S1	0.47223 (4)	0.59364 (7)	0.19075 (3)	0.02581 (11)	
O1	0.60539 (14)	0.4887 (2)	0.21492 (8)	0.0344 (3)	
O2	0.48013 (14)	0.79099 (19)	0.16197 (8)	0.0314 (3)	
N1	0.36963 (15)	0.5913 (3)	0.27530 (9)	0.0254 (3)	
H1N	0.370 (2)	0.473 (3)	0.3000 (13)	0.036 (6)*	
N2	0.19878 (19)	0.4067 (2)	0.38013 (13)	0.0310 (4)	
H2A	0.149 (3)	0.296 (4)	0.3825 (16)	0.053 (7)*	
H2B	0.228 (2)	0.440 (3)	0.4308 (15)	0.036 (6)*	
C1	0.21944 (19)	0.6768 (3)	0.27202 (12)	0.0239 (4)	
H1	0.1753 (18)	0.667 (3)	0.2111 (11)	0.020 (5)*	
C2	0.22327 (19)	0.8966 (3)	0.29742 (11)	0.0223 (4)	
H2	0.295 (2)	0.963 (3)	0.2608 (12)	0.024 (5)*	
C3	0.06161 (19)	0.9626 (2)	0.28635 (11)	0.0213 (4)	
C4	0.00777 (18)	1.1038 (3)	0.22989 (10)	0.0253 (4)	
H4	0.0742	1.1748	0.1949	0.03*	
C5	−0.1461 (2)	1.1400 (3)	0.22537 (11)	0.0276 (4)	
H5	−0.1842	1.2399	0.1886	0.033*	
C6	−0.2433 (2)	1.0319 (3)	0.27387 (12)	0.0280 (4)	
H6	−0.348	1.0544	0.2683	0.034*	
C7	−0.18973 (19)	0.8909 (3)	0.33066 (12)	0.0252 (4)	
H7	−0.257	0.8172	0.364	0.03*	
C8	−0.03595 (19)	0.8586 (2)	0.33827 (11)	0.0224 (4)	
C9	0.04146 (19)	0.7098 (3)	0.39548 (12)	0.0229 (4)	
H9	−0.0255 (19)	0.644 (3)	0.4339 (11)	0.021 (5)*	
C10	0.16898 (18)	0.8096 (2)	0.44541 (11)	0.0208 (4)	
C11	0.1950 (2)	0.8058 (3)	0.53355 (11)	0.0246 (4)	
H11	0.1301	0.7354	0.5697	0.03*	
C12	0.3175 (2)	0.9060 (3)	0.56923 (11)	0.0254 (4)	
H12	0.3357	0.9047	0.6299	0.03*	
C13	0.41273 (19)	1.0076 (3)	0.51641 (12)	0.0254 (4)	
H13	0.4947	1.0779	0.5412	0.03*	
C14	0.38929 (19)	1.0075 (3)	0.42748 (12)	0.0232 (4)	
H14	0.4565	1.0744	0.3914	0.028*	
C15	0.26676 (18)	0.9089 (2)	0.39163 (11)	0.0208 (4)	
C16	0.1156 (2)	0.5601 (3)	0.33479 (12)	0.0258 (4)	
H16	0.0421 (19)	0.500 (3)	0.2966 (11)	0.016 (4)*	
C17	0.37770 (19)	0.4609 (3)	0.10866 (11)	0.0242 (4)	
C18	0.4012 (2)	0.2615 (3)	0.10068 (12)	0.0299 (4)	
H18	0.47	0.1958	0.1383	0.036*	
C19	0.3221 (2)	0.1610 (3)	0.03665 (13)	0.0366 (5)	
H19	0.338	0.0251	0.0306	0.044*	
C20	0.2202 (2)	0.2533 (3)	−0.01897 (12)	0.0346 (5)	
C21	0.1995 (2)	0.4513 (3)	−0.00981 (12)	0.0352 (5)	
H21	0.1302	0.5167	−0.0472	0.042*	
C22	0.2777 (2)	0.5561 (3)	0.05282 (12)	0.0315 (4)	
H22	0.2632	0.6925	0.0577	0.038*	
C23	0.1345 (3)	0.1440 (4)	−0.08860 (15)	0.0525 (7)	
H23A	0.1836	0.1615	−0.1438	0.079*	
H23B	0.0319	0.1941	−0.0932	0.079*	
H23C	0.1322	0.0054	−0.074	0.079*	

### Atomic displacement parameters (Å^2^)

**Table:**

	*U*^11^	*U*^22^	*U*^33^	*U*^12^	*U*^13^	*U*^23^
S1	0.0225 (2)	0.0273 (2)	0.0275 (2)	0.0056 (2)	−0.00269 (16)	−0.0029 (2)
O1	0.0248 (7)	0.0437 (8)	0.0345 (7)	0.0115 (6)	−0.0042 (5)	−0.0040 (6)
O2	0.0285 (7)	0.0281 (7)	0.0377 (7)	−0.0004 (6)	−0.0011 (6)	−0.0022 (6)
N1	0.0255 (7)	0.0255 (8)	0.0250 (7)	0.0081 (8)	−0.0050 (6)	−0.0017 (8)
N2	0.0301 (9)	0.0185 (8)	0.0440 (11)	0.0015 (7)	−0.0045 (8)	0.0020 (8)
C1	0.0222 (9)	0.0221 (8)	0.0270 (10)	0.0063 (8)	−0.0060 (8)	−0.0029 (8)
C2	0.0213 (9)	0.0206 (9)	0.0249 (9)	0.0007 (7)	−0.0010 (7)	−0.0006 (7)
C3	0.0229 (9)	0.0188 (9)	0.0221 (9)	0.0036 (7)	−0.0037 (7)	−0.0054 (7)
C4	0.0286 (9)	0.0226 (8)	0.0244 (8)	0.0038 (9)	−0.0022 (7)	−0.0028 (9)
C5	0.0338 (10)	0.0244 (10)	0.0241 (9)	0.0094 (8)	−0.0068 (8)	−0.0024 (7)
C6	0.0243 (9)	0.0288 (10)	0.0304 (10)	0.0078 (8)	−0.0058 (8)	−0.0068 (8)
C7	0.0233 (9)	0.0224 (9)	0.0298 (10)	0.0002 (7)	−0.0024 (7)	−0.0058 (8)
C8	0.0230 (9)	0.0195 (9)	0.0242 (9)	−0.0003 (7)	−0.0053 (7)	−0.0051 (7)
C9	0.0211 (9)	0.0194 (9)	0.0283 (10)	−0.0017 (7)	0.0001 (8)	0.0016 (7)
C10	0.0199 (8)	0.0152 (8)	0.0271 (10)	0.0028 (7)	−0.0019 (7)	0.0003 (7)
C11	0.0262 (9)	0.0194 (9)	0.0283 (10)	0.0053 (7)	0.0023 (7)	0.0032 (7)
C12	0.0302 (10)	0.0229 (9)	0.0226 (9)	0.0070 (8)	−0.0061 (7)	−0.0026 (7)
C13	0.0227 (9)	0.0187 (8)	0.0343 (10)	0.0034 (7)	−0.0078 (8)	−0.0058 (8)
C14	0.0208 (9)	0.0175 (8)	0.0311 (9)	0.0022 (7)	−0.0012 (7)	−0.0003 (7)
C15	0.0198 (8)	0.0154 (8)	0.0270 (9)	0.0050 (7)	−0.0014 (7)	−0.0013 (7)
C16	0.0229 (9)	0.0193 (10)	0.0347 (10)	0.0000 (7)	−0.0070 (7)	−0.0028 (8)
C17	0.0237 (9)	0.0275 (10)	0.0216 (9)	0.0007 (8)	0.0027 (7)	−0.0010 (8)
C18	0.0323 (11)	0.0269 (10)	0.0305 (10)	0.0032 (8)	0.0039 (8)	0.0019 (9)
C19	0.0434 (12)	0.0263 (10)	0.0407 (12)	−0.0041 (9)	0.0135 (10)	−0.0067 (9)
C20	0.0315 (11)	0.0439 (12)	0.0287 (10)	−0.0074 (9)	0.0082 (8)	−0.0082 (9)
C21	0.0342 (11)	0.0448 (13)	0.0265 (10)	−0.0002 (10)	−0.0030 (8)	−0.0008 (9)
C22	0.0343 (10)	0.0309 (11)	0.0291 (10)	0.0044 (8)	−0.0022 (8)	0.0010 (8)
C23	0.0447 (12)	0.0670 (19)	0.0459 (13)	−0.0138 (12)	0.0058 (10)	−0.0235 (12)

### Geometric parameters (Å, º)

**Table:**

S1—O2	1.4309 (14)	C9—C16	1.555 (3)
S1—O1	1.4328 (13)	C9—H9	0.968 (18)
S1—N1	1.6197 (15)	C10—C11	1.380 (2)
S1—C17	1.7631 (18)	C10—C15	1.401 (2)
N1—C1	1.465 (2)	C11—C12	1.395 (3)
N1—H1N	0.90 (2)	C11—H11	0.95
N2—C16	1.460 (2)	C12—C13	1.386 (3)
N2—H2A	0.88 (3)	C12—H12	0.95
N2—H2B	0.85 (2)	C13—C14	1.389 (2)
C1—C2	1.562 (2)	C13—H13	0.95
C1—C16	1.581 (3)	C14—C15	1.391 (2)
C1—H1	1.015 (17)	C14—H14	0.95
C2—C15	1.504 (2)	C16—H16	0.967 (17)
C2—C3	1.520 (2)	C17—C22	1.391 (2)
C2—H2	0.982 (19)	C17—C18	1.393 (3)
C3—C4	1.385 (3)	C18—C19	1.388 (3)
C3—C8	1.399 (3)	C18—H18	0.95
C4—C5	1.397 (2)	C19—C20	1.390 (3)
C4—H4	0.95	C19—H19	0.95
C5—C6	1.381 (3)	C20—C21	1.382 (3)
C5—H5	0.95	C20—C23	1.508 (3)
C6—C7	1.386 (3)	C21—C22	1.384 (3)
C6—H6	0.95	C21—H21	0.95
C7—C8	1.394 (2)	C22—H22	0.95
C7—H7	0.95	C23—H23A	0.98
C8—C9	1.510 (2)	C23—H23B	0.98
C9—C10	1.524 (2)	C23—H23C	0.98
			
O2—S1—O1	120.76 (9)	C11—C10—C15	120.47 (16)
O2—S1—N1	107.20 (9)	C11—C10—C9	126.75 (16)
O1—S1—N1	105.51 (8)	C15—C10—C9	112.76 (15)
O2—S1—C17	107.00 (8)	C10—C11—C12	119.52 (17)
O1—S1—C17	107.86 (8)	C10—C11—H11	120.2
N1—S1—C17	107.96 (8)	C12—C11—H11	120.2
C1—N1—S1	120.39 (12)	C13—C12—C11	120.17 (16)
C1—N1—H1N	111.6 (13)	C13—C12—H12	119.9
S1—N1—H1N	111.1 (13)	C11—C12—H12	119.9
C16—N2—H2A	113.1 (16)	C12—C13—C14	120.51 (16)
C16—N2—H2B	113.2 (16)	C12—C13—H13	119.7
H2A—N2—H2B	109 (2)	C14—C13—H13	119.7
N1—C1—C2	111.46 (14)	C13—C14—C15	119.52 (17)
N1—C1—C16	109.09 (14)	C13—C14—H14	120.2
C2—C1—C16	110.19 (15)	C15—C14—H14	120.2
N1—C1—H1	109.8 (10)	C14—C15—C10	119.77 (16)
C2—C1—H1	107.7 (10)	C14—C15—C2	126.55 (16)
C16—C1—H1	108.6 (10)	C10—C15—C2	113.69 (15)
C15—C2—C3	108.25 (14)	N2—C16—C9	113.99 (15)
C15—C2—C1	107.61 (14)	N2—C16—C1	111.32 (14)
C3—C2—C1	104.26 (14)	C9—C16—C1	107.66 (14)
C15—C2—H2	112.3 (11)	N2—C16—H16	108.2 (11)
C3—C2—H2	115.5 (11)	C9—C16—H16	111.2 (10)
C1—C2—H2	108.3 (11)	C1—C16—H16	104.0 (10)
C4—C3—C8	120.72 (16)	C22—C17—C18	120.25 (17)
C4—C3—C2	126.44 (16)	C22—C17—S1	119.53 (14)
C8—C3—C2	112.80 (15)	C18—C17—S1	120.20 (14)
C3—C4—C5	118.77 (17)	C19—C18—C17	118.60 (18)
C3—C4—H4	120.6	C19—C18—H18	120.7
C5—C4—H4	120.6	C17—C18—H18	120.7
C6—C5—C4	120.62 (16)	C18—C19—C20	121.91 (18)
C6—C5—H5	119.7	C18—C19—H19	119.0
C4—C5—H5	119.7	C20—C19—H19	119.0
C5—C6—C7	120.72 (17)	C21—C20—C19	118.26 (18)
C5—C6—H6	119.6	C21—C20—C23	119.9 (2)
C7—C6—H6	119.6	C19—C20—C23	121.9 (2)
C6—C7—C8	119.20 (17)	C20—C21—C22	121.26 (19)
C6—C7—H7	120.4	C20—C21—H21	119.4
C8—C7—H7	120.4	C22—C21—H21	119.4
C7—C8—C3	119.86 (16)	C21—C22—C17	119.70 (18)
C7—C8—C9	126.36 (16)	C21—C22—H22	120.2
C3—C8—C9	113.69 (15)	C17—C22—H22	120.2
C8—C9—C10	108.52 (14)	C20—C23—H23A	109.5
C8—C9—C16	106.81 (14)	C20—C23—H23B	109.5
C10—C9—C16	106.30 (14)	H23A—C23—H23B	109.5
C8—C9—H9	113.4 (10)	C20—C23—H23C	109.5
C10—C9—H9	111.4 (10)	H23A—C23—H23C	109.5
C16—C9—H9	110.0 (11)	H23B—C23—H23C	109.5
			
O2—S1—N1—C1	53.66 (16)	C12—C13—C14—C15	1.8 (3)
O1—S1—N1—C1	−176.43 (14)	C13—C14—C15—C10	−0.4 (2)
C17—S1—N1—C1	−61.31 (17)	C13—C14—C15—C2	178.89 (16)
S1—N1—C1—C2	−90.01 (17)	C11—C10—C15—C14	−1.3 (2)
S1—N1—C1—C16	148.09 (14)	C9—C10—C15—C14	−179.93 (15)
N1—C1—C2—C15	−67.85 (18)	C11—C10—C15—C2	179.27 (15)
C16—C1—C2—C15	53.41 (18)	C9—C10—C15—C2	0.7 (2)
N1—C1—C2—C3	177.35 (14)	C3—C2—C15—C14	−126.01 (18)
C16—C1—C2—C3	−61.39 (17)	C1—C2—C15—C14	121.86 (18)
C15—C2—C3—C4	127.49 (18)	C3—C2—C15—C10	53.35 (19)
C1—C2—C3—C4	−118.15 (18)	C1—C2—C15—C10	−58.78 (18)
C15—C2—C3—C8	−54.95 (19)	C8—C9—C16—N2	178.79 (14)
C1—C2—C3—C8	59.40 (18)	C10—C9—C16—N2	63.07 (19)
C8—C3—C4—C5	−0.3 (2)	C8—C9—C16—C1	54.78 (16)
C2—C3—C4—C5	177.07 (16)	C10—C9—C16—C1	−60.94 (17)
C3—C4—C5—C6	−2.4 (3)	N1—C1—C16—N2	2.1 (2)
C4—C5—C6—C7	2.7 (3)	C2—C1—C16—N2	−120.58 (16)
C5—C6—C7—C8	−0.2 (3)	N1—C1—C16—C9	127.70 (15)
C6—C7—C8—C3	−2.6 (3)	C2—C1—C16—C9	5.03 (18)
C6—C7—C8—C9	−178.97 (16)	O2—S1—C17—C22	−25.90 (17)
C4—C3—C8—C7	2.8 (3)	O1—S1—C17—C22	−157.23 (14)
C2—C3—C8—C7	−174.90 (15)	N1—S1—C17—C22	89.20 (16)
C4—C3—C8—C9	179.66 (15)	O2—S1—C17—C18	155.12 (14)
C2—C3—C8—C9	1.9 (2)	O1—S1—C17—C18	23.79 (17)
C7—C8—C9—C10	−131.40 (18)	N1—S1—C17—C18	−89.78 (16)
C3—C8—C9—C10	52.0 (2)	C22—C17—C18—C19	−0.5 (3)
C7—C8—C9—C16	114.37 (19)	S1—C17—C18—C19	178.43 (13)
C3—C8—C9—C16	−62.23 (18)	C17—C18—C19—C20	−0.3 (3)
C8—C9—C10—C11	127.94 (18)	C18—C19—C20—C21	0.6 (3)
C16—C9—C10—C11	−117.50 (18)	C18—C19—C20—C23	179.92 (19)
C8—C9—C10—C15	−53.57 (19)	C19—C20—C21—C22	0.1 (3)
C16—C9—C10—C15	60.99 (18)	C23—C20—C21—C22	−179.28 (19)
C15—C10—C11—C12	1.8 (2)	C20—C21—C22—C17	−0.9 (3)
C9—C10—C11—C12	−179.86 (16)	C18—C17—C22—C21	1.2 (3)
C10—C11—C12—C13	−0.4 (3)	S1—C17—C22—C21	−177.80 (15)
C11—C12—C13—C14	−1.3 (3)		

### Hydrogen-bond geometry (Å, º)

Cg1 is the centroid of the C3–C8 benzene ring.

**Table:**

*D*—H···*A*	*D*—H	H···*A*	*D*···*A*	*D*—H···*A*
N1—H1*N*···N2	0.90 (2)	2.04 (2)	2.592 (2)	117.9 (17)
C11—H11···*Cg*1^i^	0.95	2.87	3.712 (2)	149
C5—H5···O1^ii^	0.95	2.58	3.269 (2)	129
C12—H12···O1^iii^	0.95	2.52	3.446 (2)	166
C23—H23*A*···O1^iv^	0.98	2.51	3.259 (3)	133

Symmetry codes: (i) −*x*, *y*−1/2, −*z*+1; (ii) *x*−1, *y*+1, *z*; (iii) −*x*+1, *y*+1/2, −*z*+1; (iv) −*x*+1, *y*−1/2, −*z*.
